# A phenomenological approach to the affective core of Delusional Disorder.

**DOI:** 10.1192/j.eurpsy.2023.2254

**Published:** 2023-07-19

**Authors:** R. Pellegrini, J. E. Muñoz Negro, R. Ottoni, M. Tonna, J. A. Cervilla

**Affiliations:** 1 Psychiatry, Hospital Universitario de Cruces, Barakaldo; 2 Psychiatry, Universidad de Granada, Granada, Spain; 3 psychiatry, University of Parma, Parma, Italy

## Abstract

**Introduction:**

According to current diagnostic systems, affective symptoms do not represent a fundamental criterion for the diagnosis of DD. However, numerous studies have highlighted frequent comorbidity between DD and Mood Disorders and have elucidated the importance of the affective state in the development and persistence of delusions. Thus, some factor analysis studies have identified the existence of a depressive dimension in DD, suggesting a substantial psychopathological heterogeneity in DD. However, these important affective features have not evaluated from a phenomenological point of view and in their relationship with delusions.

**Objectives:**

The aim of the present study is to investigate the relationship between personality, trait affectivity and severity of delusions in patients with Delusional Disorder (DD).

**Methods:**

Thirty-two outpatients affected by DD were administered the Structured Interview for DSM-IV-TR Personality Disorders (SIDP-IV), the Pathological Narcissism Inventory (NPI), the Positive and Negative Affect Schedule (PANAS) and the Psychotic Symptom Rating Scale (PSYRATS). Next, we analysed the prevalence of personality disorder in our sample of patients with DD and studied the correlations between the severity of delusions and the different affective variables. Finally, we obtained a multivariate explanatory model of the severity of the delusions.

**Results:**

The severity of delusions was directly associated with “Grandiose Fantasy” item of narcissistic personality and inversely related with the feelings of shame, fear and guilt. In the multivariate model, the feeling of shame was the only independent variable capable of accounting for the severity of the delusions, that, in in DD patients, would lie on an affective core of shame.

**Table 2.** Pearson correlation coefficient

VARIABLES	DRS total score
**GRANDIOSE FANTASY**	**p = 0.045**
**SHAME**	**p = 0.048**
**GUILT**	**p = 0.016**
AGITATION	p = 0.049
FEAR	p = 0.041

**Table 3.** Standardized coefficients in linear standard regression (DRS)

	DRS: total score			
	R²	β	t	p
*Step 1*	0.157	-	-	**-**
**GRANDIOSE FANTASY**	-	0.396	1.89	**0.048**
*Step 2*	0.460	-	-	**-**
**GRANDIOSE FANTASY**	-	0.291	1.65	**0.116**
**SHAME**	-	-0.560	-3.17	**0.005**

**Conclusions:**

The severity of delusional beliefs in DD patients would lie on an affective core of shame upon predisposing personality traits. These findings could help to develop a psychotherapeutic approach for delusional patients focused in the feeling of shame.

**Disclosure of Interest:**

None Declared

